# Integrative omics and multi-cohort identify *IRF1* and biological targets related to sepsis-associated acute respiratory distress syndrome

**DOI:** 10.7555/JBR.39.20250066

**Published:** 2025-05-27

**Authors:** Jiajin Chen, Ruili Hou, Xiaowen Xu, Ning Xie, Jiaqi Tang, Yi Li, Xiaoqing Nie, Nuala J. Meyer, Li Su, David C. Christiani, Feng Chen, Ruyang Zhang

**Affiliations:** 1 Institute of Cardiovascular Diseases, Xiamen Cardiovascular Hospital of Xiamen University, Xiamen University School of Medicine, Xiamen, Fujian 361006, China; 2 Department of Biostatistics, Center for Global Health, School of Public Health, Nanjing Medical University, Nanjing, Jiangsu 211166, China; 3 China International Cooperation Center for Environment and Human Health, Nanjing Medical University, Nanjing, Jiangsu 211166, China; 4 Department of Biostatistics, University of Michigan, Ann Arbor, MI 48109, USA; 5 Pulmonary, Allergy, and Critical Care Medicine Division, University of Pennsylvania Perelman School of Medicine, Philadelphia, PA 19104, USA; 6 Department of Environmental Health, Harvard T.H. Chan School of Public Health, Boston, MA 02115, USA; 7 Pulmonary and Critical Care Division, Department of Medicine, Massachusetts General Hospital and Harvard Medical School, Boston, MA 02114, USA; 8 Changzhou Medical Center, Nanjing Medical University, Changzhou, Jiangsu 213164, China

**Keywords:** acute respiratory distress syndrome, sepsis, interferon regulatory factor 1, causal inference, immunity

## Abstract

Interferon-related genes are involved in antiviral responses, inflammation, and immunity, which are closely related to sepsis-associated acute respiratory distress syndrome (ARDS). We analyzed 1972 participants with genotype data and 681 participants with gene expression data from the Molecular Epidemiology of ARDS (MEARDS), the Molecular Epidemiology of Sepsis in the ICU (MESSI), and the Molecular Diagnosis and Risk Stratification of Sepsis (MARS) cohorts in a three-step study focusing on sepsis-associated ARDS and sepsis-only controls. First, we identified and validated interferon-related genes associated with sepsis-associated ARDS risk using genetically regulated gene expression (GReX). Second, we examined the association of the confirmed gene (interferon regulatory factor 1, *IRF1*) with ARDS risk and survival and conducted a mediation analysis. Through discovery and validation, we found that the GReX of *IRF1* was associated with ARDS risk (odds ratio [OR_MEARDS_] = 0.84, *P* = 0.008; OR_MESSI_ = 0.83, *P* = 0.034). Furthermore, individual-level measured *IRF1* expression was associated with reduced ARDS risk (OR = 0.58, *P* = 8.67 × 10^−4^), and improved overall survival in ARDS patients (hazard ratio [HR_28-day_] = 0.49, *P* = 0.009) and sepsis patients (HR_28-day_ = 0.76, *P* = 0.008). Mediation analysis revealed that *IRF1* may enhance immune function by regulating the major histocompatibility complex, including *HLA-F*, which mediated more than 70% of protective effects of *IRF1* on ARDS. The findings were validated by *in vitro* biological experiments including time-series infection dynamics, overexpression, knockout, and chromatin immunoprecipitation sequencing. Early prophylactic interventions to activate *IRF1* in sepsis patients, thereby regulating *HLA-F*, may reduce the risk of ARDS and mortality, especially in severely ill patients.

## Introduction

Acute respiratory distress syndrome (ARDS), a form of acute hypoxemic respiratory failure primarily induced by sepsis and accompanied by severe inflammatory pulmonary edema^[1]^, is associated with high morbidity and mortality in intensive care units (ICUs)^[2]^. Despite decades of research, there are few effective pharmacotherapies available for sepsis-associated ARDS, and the in-hospital mortality rate remains above 40%^[3]^, causing a severe global health concern.

Interferon (IFN)-related genes play essential roles in immunity and inflammation^[4]^. Some preclinical studies have demonstrated their preventive and therapeutic effects on coronavirus-induced ARDS, as well as other types of ARDS^[5–10]^. Additionally, *in vitro* studies have shown that IFNs improve host defenses and inhibit the replication of coronaviruses^[5–7]^. *In vivo* animal models have also demonstrated the benefits of IFN therapy in reducing ARDS severity and prolonging survival^[8–9]^. Moreover, emerging evidence suggests the crucial roles of IFN regulatory factors^[11–12]^, IFN receptors^[13]^, and IFN-stimulated genes^[14]^ in both independently activating immune responses and directly engaging in antiviral processes^[14–15]^. However, clinical trials in ARDS patients have reported inconsistent results^[11,16]^, possibly because of heterogeneous populations, limited sample sizes, and a lack of causal inference-based investigations.

Causal biomarkers identified in large population cohorts are often considered promising and valuable therapeutic targets. To investigate causal relationships in large-scale populations, transcriptome-wide association studies (TWAS) use genetic variants to estimate genetically regulated gene expression (GReX) *via* integrative analysis of genotype data and expression quantitative trait loci (eQTLs), followed by the evaluation of causal associations between GReX and traits^[17–19]^. TWAS enables adjustment for unmeasured confounders while estimating causal effects, making it a powerful approach in various disease studies^[20]^.

In the present study, based on trans-omics data from the Molecular Epidemiology of ARDS (MEARDS), the Molecular Epidemiology of Sepsis in the ICU (MESSI), and the Molecular Diagnosis and Risk Stratification of Sepsis (MARS) cohorts, we conducted a three-step study with multiple cohorts and biological experiments and employed causal inference to evaluate the causal effects and the potential pathogenic and prognostic pathways of IFN-related genes on sepsis-associated ARDS.

## Subjects and methods

### Study participants and trans-omics data from three independent cohorts

This study was part of the original study titled MEARDS and was approved by the Institutional Review Boards (IRBs; Approval No. 1999P0086071/MGH) of the Harvard T.H. Chan School of Public Health, Massachusetts General Hospital (MGH), and Beth Israel Deaconess Medical Center (BIDMC) in 1999. Written informed consent was obtained from all participants or their surrogates. The MESSI (Approval No. 808542) and MARS (Approval No. 10-056C) cohorts were approved by their respective IRBs.

Participants in the MEARDS study were recruited from the ICUs of MGH and BIDMC between 1998 and 2014 (ClinicalTrials.gov: NCT00006496)^[21–24]^. Eligible participants were critically ill patients with at least one predisposing condition for ARDS and without any exclusion criteria. The MESSI cohort was a prospective study conducted in the medical ICU of the Hospital of the University of Pennsylvania, an urban tertiary referral center, between 2008 and 2015^[25]^. Participants were eligible if they were admitted with sepsis, and were excluded if an alternative diagnosis explained the systemic inflammatory response syndrome, if they were declining life support on admission, or if they were unable to provide informed consent. The MARS cohort was a prospective observational study conducted in the mixed ICUs of two tertiary teaching hospitals (Academic Medical Center in Amsterdam and University Medical Center in Utrecht), between January 2011 and July 2013^[26]^. All consecutive patients older than 18 years of age admitted during this period, with an expected length of stay longer than 24 h, were included. More details are provided in ***Supplementary Methods***. All study methodologies conformed to the standards set by the 1975 Declaration of Helsinki.

The MEARDS genome-wide association study (GWAS) dataset was deposited in dbGaP under accession code phs000631.v1.p1. For the MESSI GWAS, dbGaP submission is forthcoming in accordance with the NIH genomic data-sharing policy. The MEARDS RNA-seq dataset is available upon request from the authors. The MARS RNA-array dataset is available in GEO under accession code GSE65682. Time-series RNA expression data from sepsis patients and cell models were deposited in datasets with accession numbers GSE54514 and GSE146532, respectively. Data for cell overexpression, knockout, and chromatin immunoprecipitation sequencing (ChIP-seq) experiments were downloaded from datasets with accession numbers GSE181861, GSE114284, and GSE100381, respectively.

Considering the heterogeneity between ARDS subtypes, the current study focused on the main subtype of ARDS, namely, sepsis-associated ARDS and sepsis-only controls. Only participants of European ancestry were retained for the analyses, because genetic differences exist among ethnicities and few TWAS models are available for non-European populations. Moreover, to ensure comparability between the MEARDS and MESSI cohorts, we harmonized the ARDS definition in accordance with the Berlin diagnostic criteria (including arterial blood gas, chest X-ray criteria, onset within one week, *etc.*)^[27–31]^ and implemented standard imputation and quality control processes for both cohorts. Information regarding genotyping, variant imputation, sequencing, and quality control procedures is detailed in the ***Supplementary Methods***. Finally, we included 1369 and 603 participants with genotype data from the MEARDS and MESSI cohorts, and 213 and 468 participants with gene expression data from the MEARDS and MARS cohorts, respectively (***Supplementary Fig. 1***). Population characteristics are detailed in ***Supplementary Table 1***.

### Definition of IFN-related gene

The IFN-related gene families were identified according to the HUGO Gene Nomenclature Committee (HGNC; https://www.genenames.org/). We identified 64 IFN-related genes belonging to five distinct IFN-related gene families, as detailed in ***Supplementary Table 2***.

### Transcriptome-wide association study

TWAS evaluates the causal relationship between gene expression and traits using a two-step framework of Mendelian randomization. In the current study, we performed TWAS analysis using the multivariate adaptive shrinkage (MASHR) method^[13,18–19]^ (recommended by the PrediXcan team), which jointly constructs TWAS models across multiple tissues, leveraging correlations across tissues to increase power, identify tissue-specific and shared eQTLs, and improve the accuracy of estimated eQTL effects^[19]^ (***Supplementary Methods***). Specifically, we obtained the MASHR models, which were pre-constructed by the PrediXcan team, from the PredictDB database (http://www.predictdb.org/). The GReX of IFN-related genes was predicted under the PrediXcan framework^[17]^. Additionally, the causal associations between the GReX of IFN-related genes and ARDS risk were assessed by logistic regression models.

### Study design

**Figure 1:**
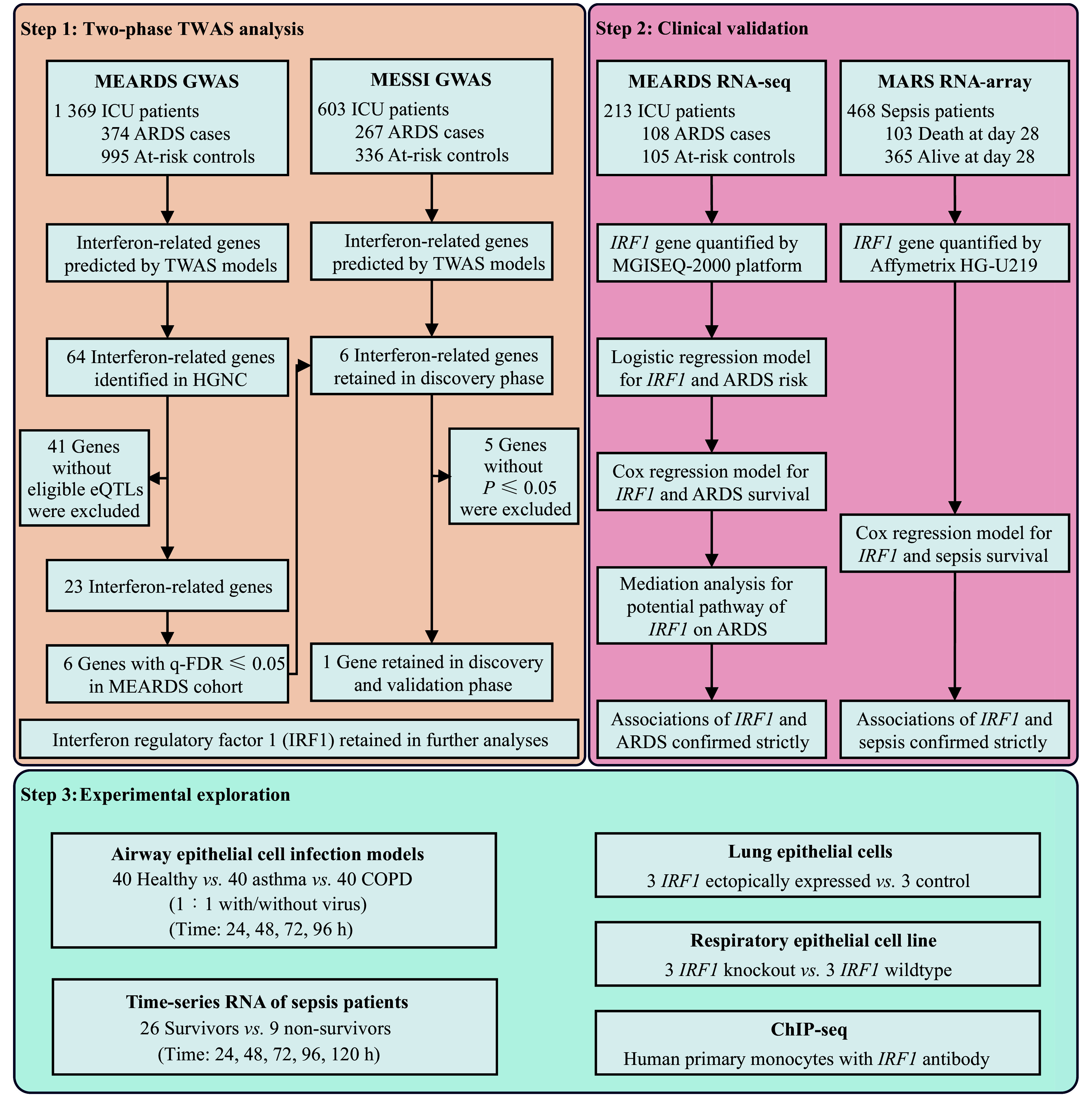
Flowchart of a three-step analytic strategy to screen and validate sepsis-associated ARDS-related interferon (IFN)-related genes. The first step is a two-phase TWAS analysis. The causal effects of IFN-related genes on ARDS risk were discovered and validated in MEARDS and MESSI cohorts. In the clinical validation step, the associations of the robustly confirmed gene (*IRF1*) with ARDS risk and survival, as well as sepsis survival, were examined using the MEARDS RNA-seq and MARS RNA-array datasets, respectively. In the experimental exploration step, *in vitro* biological experiments regarding time-series infection dynamics, overexpression, knockout, and chromatin immunoprecipitation sequencing were used to verify the findings. Abbreviations: ARDS, acute respiratory distress syndrome; COPD, chronic obstructive pulmonary disease; eQTLs, expression quantitative trait loci; GWAS, genome-wide association study; HGNC, HUGO Gene Nomenclature Committee; ICU, intensive care unit; MARS, Molecular Diagnosis and Risk Stratification of Sepsis; MEARDS, Molecular Epidemiology of ARDS; MESSI, Molecular Epidemiology of Sepsis in the ICU; TWAS, transcriptome-wide association study.

***Figure 1*** shows a three-step study design. Step 1 is a two-phase TWAS analysis. Based on the MEARDS genotype dataset, we predicted the genetically regulated expression of IFN-related genes using MASHR-based TWAS models and identified ARDS risk-associated genes using logistic regression models. Then, we validated the identified gene GReX (*q*-FDR ≤ 0.05) using an independent MESSI genotype dataset through TWAS analysis. Candidate genes were identified if they met *P* ≤ 0.05 and had consistent directions of effect in the two cohorts. Finally, *IRF1* alone was identified and validated as a candidate gene for further clinical validation.

In the clinical validation step, we verified the associations between measured *IRF1* expression and ARDS risk using a logistic regression model based on the MEARDS RNA-seq dataset. Moreover, the associations of *IRF1* expression at ICU recruitment with the overall survival of ARDS patients and sepsis patients were evaluated using Cox regression models based on the MEARDS and MARS RNA datasets. Finally, we investigated the potential downstream pathogenic and prognostic mediators of *IRF1* using VanderWeele's mediation analysis.

In the experimental exploration step, we verified the associations between *IRF1*, *HLA-F*, and respiratory diseases using airway epithelial cell infection models. Cells collected from 40 healthy participants, 40 patients with asthma, and 40 patients with chronic obstructive pulmonary disease (COPD) were cultured 1∶1 with or without rhinovirus 1A and harvested at four time points (24, 48, 72, and 96 h) for mRNA expression measurement. Moreover, the observed results were further verified in 35 sepsis patients (26 survivors and nine non-survivors) with time-series RNA data collected daily for up to five days. Additionally, the regulatory relationship between *IRF1* and *HLA-F* was experimentally validated by ectopic expression experiments (lung epithelial cells, three *IRF1* ectopically expressed *vs.* three control), knockout experiments (respiratory epithelial cell line, three *IRF1* knockout *vs.* three *IRF1* wild-type samples), and ChIP-seq; the experimental populations and methods have been described previously^[32–34]^.

### Statistical analysis

Continuous variables were summarized as mean ± standard deviations, and categorical variables were described as frequency (*n*) and proportion (%). The Acute Physiology and Chronic Health Evaluation (APACHE) Ⅲ score was used to describe the degree of physiologic severity. PhenomeXcan was employed to evaluate the phenome-wide associations of *IRF1* with 4049 traits in the UK Biobank^[35]^. Pathway activity and immune cell types were identified through single-sample gene set enrichment analysis (ssGSEA), and the gene network was assessed using GeneMANIA. In the analysis of GWAS and RNA-seq data in the MEARDS cohort, the covariates adjusted for in the models included age, sex, pneumonia, transfer, multiple transfusions, trauma, and aspiration for risk analysis, with the additional inclusion of the APACHE score for survival analysis. Because of data availability, we adjusted for age, sex, pneumonia, and APACHE Ⅲ in the MESSI cohort, and age, sex, and pneumonia in the MARS cohort. Statistical analyses were conducted using R version 3.6.3, and a two-sided *P*-value ≤ 0.05 was considered statistically significant unless otherwise specified.

## Results

### Two-phase TWAS analysis discovered and validated *IRF1* as a potential causal gene for sepsis-associated ARDS risk

**Figure 2:**
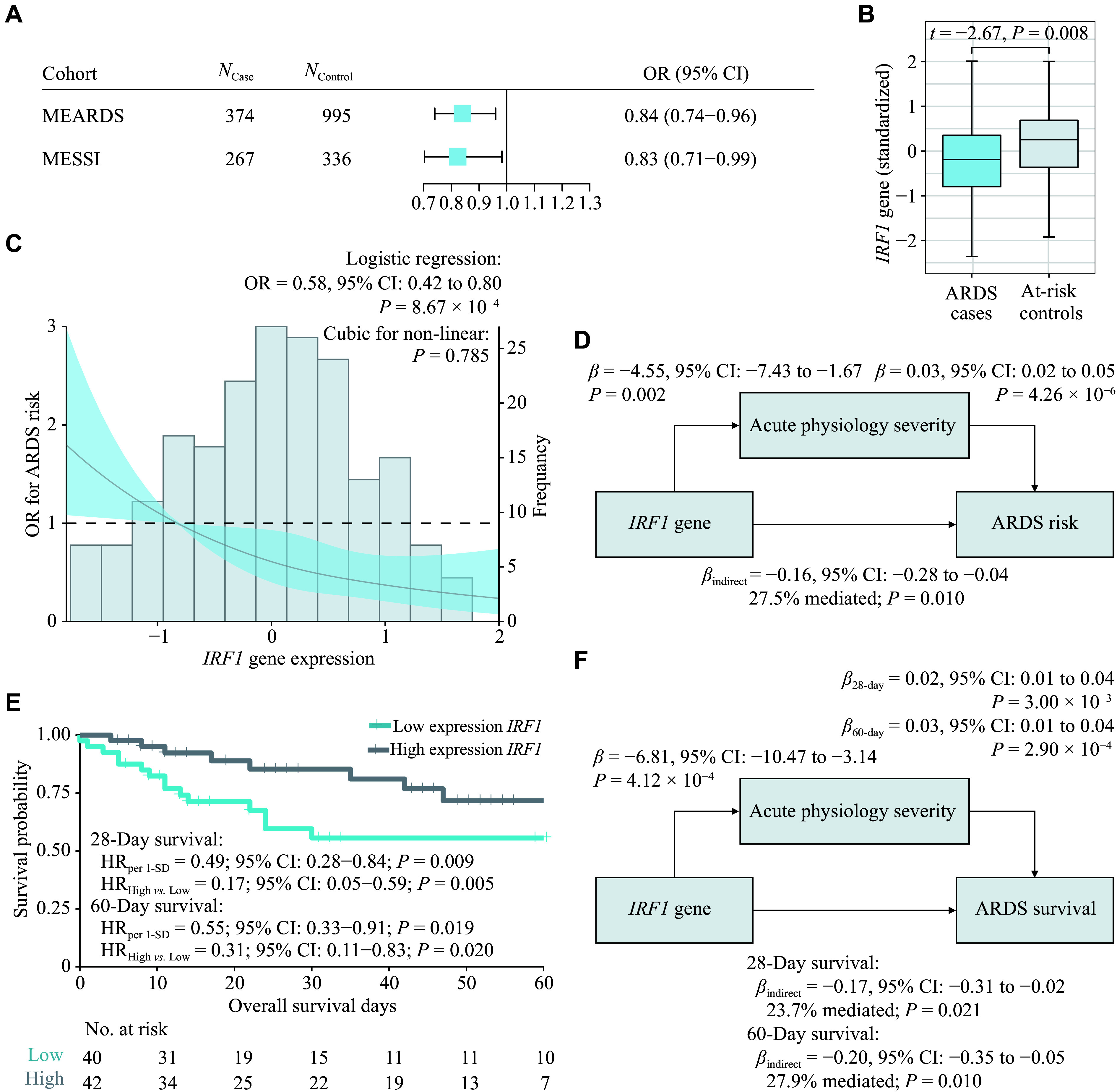
Association between *IRF1*, acute physiology severity, ARDS risk and survival. A: Forest plot showing associations between GReX of *IRF1* and ARDS risk in the MEARDS and MESSI cohorts. B: Distribution of measured *IRF1* expression in ARDS cases (*n* = 108) and at-risk controls (*n* = 105). C: Odds ratio (OR) of *IRF1* expression on ARDS risk and non-linear association explored by spline regression. D: Mediation analysis for the indirect effect of *IRF1* on ARDS risk through acute physiology severity. E: Kaplan–Meier survival curves for the survival difference between ARDS patients with low and high expression of *IRF1* (classified by the median value of 7.119). F: Mediation analysis for the indirect effect of *IRF1* on ARDS 28-day and 60-day survival *via* acute physiology severity. Abbreviations: CI, confidence interval; IRF1, interferon regulatory factor 1; MEARDS, Molecular Epidemiology of ARDS; MESSI, Molecular Epidemiology of Sepsis in the ICU.

Among the 64 IFN-related genes annotated in the HGNC database, 23 genes with eligible MASHR models (requiring robust cross-tissue eQTL data for GReX prediction) were included in the analysis, while 41 genes were excluded due to insufficient eQTL evidence (*e.g.*, lack of significant *cis*-eQTLs or tissue-specific models). In the discovery phase, we employed logistic regression models to evaluate the associations between the GReX of IFN-related genes and ARDS risk based on the MEARDS genotype database. This stringent filtering ensured methodological rigor, as TWAS relies on high-confidence eQTL models to infer causal relationships between gene expression and phenotypic traits. Six genes showed a false discovery rate (FDR)-adjusted *q*-value (*q*-FDR) ≤ 0.05 (***Supplementary Table 3***). Among them, *IRF1* was confirmed with both statistical significance and a consistent direction of effect when validated using the MESSI genotype database in the validation phase (odds ratio [OR_MEARDS_] = 0.84, 95% confidence interval [CI]: 0.74 to 0.96, *P =* 0.008; OR_MESSI_ = 0.83, 95% CI: 0.71 to 0.99, *P =* 0.034; ***Fig. 2A*** and ***Supplementary Table 3***).

### Clinical validation with measured gene expression data demonstrated the *IRF1*-ARDS associations, which were mediated by acute physiologic severity

In the clinical validation step, we compared *IRF1* gene expression levels between ARDS cases and non-ARDS controls based on individual-level measured gene expression from the MEARDS RNA-seq dataset. The expression level of *IRF1*, presented as the mean ± standard deviation, was significantly higher in the control group (0.20 ± 0.97) than in the case group (−0.21 ± 0.99; *t* = −2.67, *P* = 0.008) (***Fig. 2B***). The association was further demonstrated by a covariate-adjusted logistic regression model (OR *=* 0.58, 95% CI: 0.42 to 0.80, *P =* 8.67×10^−4^). However, a spline regression model revealed no evidence of non-linear relationship between *IRF1* expression and ARDS risk (*P =* 0.785; ***Fig. 2C***). Notably, higher *IRF1* expression levels were significantly associated with less severe acute physiological status (*β =* −4.55, 95% CI: −7.43 to −1.67, *P =* 0.002; ***Fig. 2D***). Mediation analysis further revealed that approximately 27.5% of the protective effect of *IRF1* on ARDS risk was mediated by attenuation of acute physiology severity (*β*_indirect_ = −0.16, 95% CI: −0.28 to −0.04, *P =* 0.010; ***Fig. 2D***). We further evaluated the correlation between TWAS-predicted and actual RNA-seq-measured *IRF1* expression in samples with both GWAS and RNA-seq data, and observed a weak correlation (*r* = 0.105, *P* = 0.46), which might be due to the small sample size and limited genetic heritability.

### Acute physiologic severity mediated the protective effect of *IRF1* on sepsis-associated ARDS survival

We observed a protective effect of *IRF1* on 28- and 60-day survival in ARDS patients (hazard ratio [HR_28-day_] = 0.49, 95% CI: 0.28 to 0.84, *P =* 0.009; HR_60-day_ = 0.55, 95% CI: 0.33 to 0.91, *P =* 0.019). Patients were stratified into high- and low-expression groups based on the median *IRF1* expression level (7.119) in the MEARDS RNA-seq cohort. Compared with those with lower *IRF1* expression levels, patients with higher *IRF1* expression levels had improved survival rates at 28 days (HR_High *vs.* Low_ = 0.17, 95% CI: 0.05 to 0.59, *P =* 0.005) and 60 days (HR_High *vs.* Low_ = 0.31, 95% CI: 0.11 to 0.83, *P =* 0.020; ***Fig. 2E***). Moreover, the effects of *IRF1* on 28-day survival (*β*_indirect_ = −0.17, 95% CI: −0.31 to −0.02, 23.7% mediated, *P =* 0.021) and 60-day survival (*β*_indirect_ = −0.20, 95% CI: −0.35 to −0.05, 27.9% mediated, *P =* 0.010) of ARDS patients were significantly mediated by attenuated acute physiology severity (***Fig. 2F***). Additionally, a significant protective effect of *IRF1* on the survival of sepsis patients was also observed in the MARS cohort (HR_per 1-SD_ = 0.76, 95% CI: 0.62 to 0.93, *P =* 0.008; HR_High *vs.* Low_ = 0.64, 95% CI: 0.44 to 0.95, *P =* 0.025; ***Supplementary Fig. 2***).

### *In silico* analyses revealed the potential functions of *IRF1* in immunity

**Figure 3:**
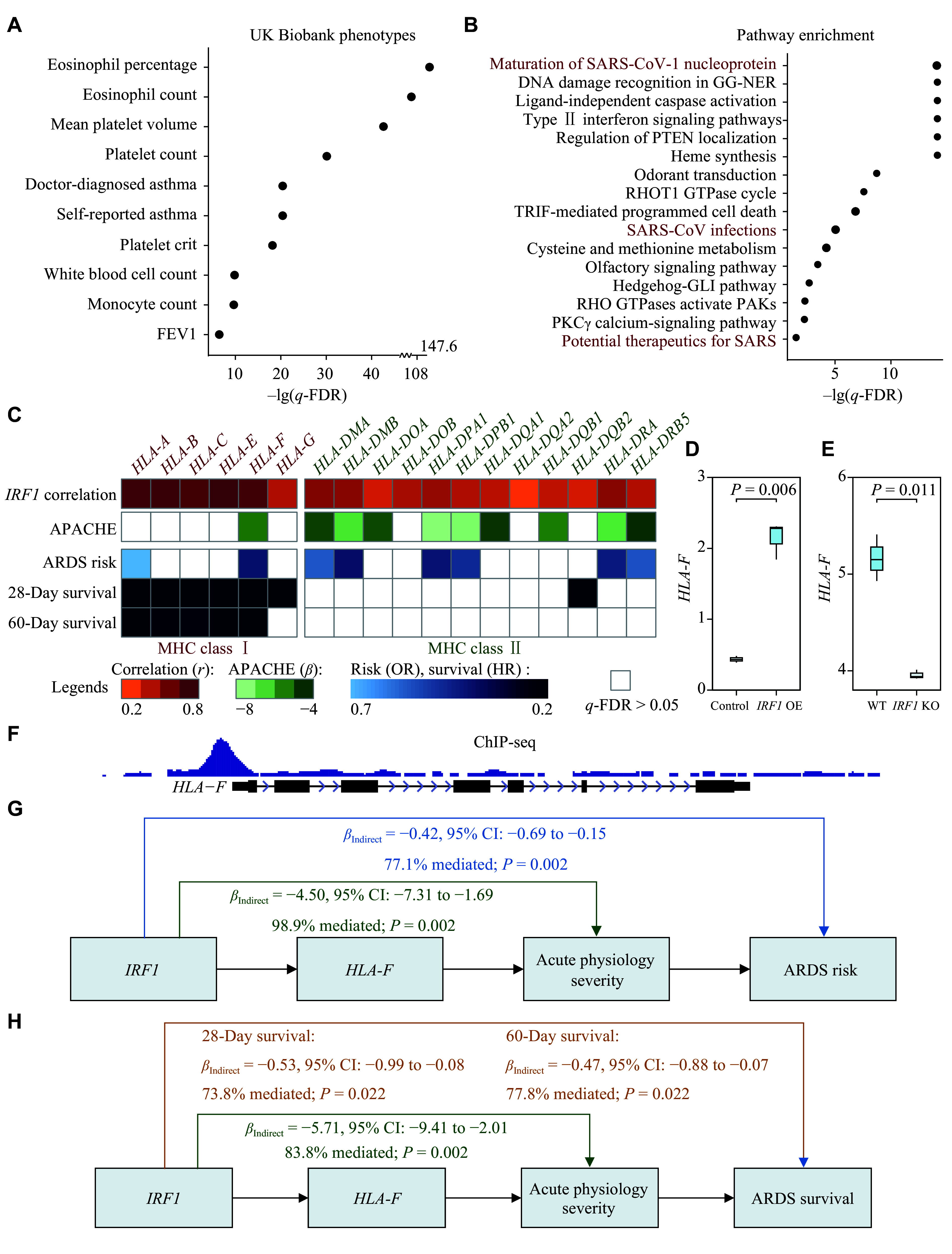
Biologic functions of *IRF1* and its potential downstream target on MHC. A: Phenome-wide association study for *IRF1* and 4049 traits in the UK Biobank. B: Sixteen *IRF1*-correlated pathways associated with both ARDS risk and survival were identified through single-sample gene set enrichment analysis. C: Heatmap showing correlations among MHC genes, *IRF1* expression, acute physiology severity, ARDS risk, and survival, analyzed using MEARDS-seq data. D: *IRF1* overexpression experiment in lung epithelial cells from GSE181861. E: *IRF1* knockout experiment in respiratory epithelial cells (GSE114284). F: ChIP-seq experiment revealed a binding between *IRF1* and *HLA-F* (GSE100381). G–H: Mediation analyses for the indirect effects of *IRF1* regulating *HLA-F* on acute physiology severity, ARDS risk, and 28-day and 60-day survival of ARDS patients, analyzed using MEARDS-seq data. Abbreviations: APACHE, acute physiology and chronic health evaluation; ARDS, acute respiratory distress syndrome; CI, confidence interval; HLA-F, human leukocyte antigen F; FDR, false discovery rate; HR, hazard ratio; IRF1, interferon regulatory factor 1; KO, knockout; MEARDS, Molecular Epidemiology of ARDS; OE, overexpression; OR, odds ratio; WT, wild-type.

Based on the PhenomeXcan platform, we observed that *IRF1* was significantly associated with ARDS-related traits in the UK Biobank (*q*-FDR ≤ 0.05), including eosinophil, platelet, white blood cell, monocyte, asthma, and lung function (***Fig. 3A*** and ***Supplementary Fig. 3***). Furthermore, ssGSEA analysis revealed a total of 1127 upregulated and 219 downregulated pathways that were correlated with *IRF1* (***Supplementary Fig. 4***). Of these, 16 *IRF1*-correlated pathways were associated with both ARDS risk and patient survival (***Fig. 3B***; ***Supplementary Table 4***); notably, three of these pathways were related to SARS-CoV infection or therapy (***Fig. 3B***). Moreover, based on ssGSEA-inferred immune cell abundances, 10 out of the 28 immune cell types showed significant correlations with *IRF1* expression (***Supplementary Fig. 5***). The gene network also revealed potential functional connections between *IRF1* and immune checkpoint genes (***Supplementary Fig. 6***). Additionally, numerous immunity-related drugs targeting *IRF1* have been documented in the DrugBank database (***Supplementary Table 5***).

### *IRF1* regulated major histocompatibility complex (MHC) to affect sepsis-associated ARDS

As suggested by *in silico* bioinformatic analysis, *IRF1* may play critical roles in antiviral immune responses. We therefore explored the potential immune pathway mediating the *IRF1*-ARDS associations. Given that the MHC is a well-established component of immune responses, we included it as a potential mediator in the mediation analysis and observed significant co-expression of *IRF1* with both MHC class Ⅰ (*r*_min_ = 0.40 to *r*_max_ = 0.89) and MHC class Ⅱ genes (*r*_min_ = 0.15 to *r*_max_ = 0.55; ***Supplementary Fig. 7***). The MHC class Ⅰ genes tended to be associated with ARDS survival, whereas the MHC class Ⅱ genes were more likely to affect ARDS risk (***Fig. 3C***). In particular, *HLA-F*, an MHC class Ⅰ gene, was significantly associated with all outcomes, including acute physiological severity (*β =* −5.23, 95% CI: −8.42 to −2.04, *P =* 0.002), ARDS risk (OR *=* 0.58, 95% CI: 0.42 to 0.80, *P =* 0.001), and both 28- and 60-day survival of ARDS patients (HR_28-day_ = 0.41, 95% CI: 0.24 to 0.69, *P =* 8.23×10^−4^; HR_60-day_ = 0.48, 95% CI: 0.30 to 0.77, *P =* 0.002) (***Fig. 3C***; ***Supplementary Table 6***). The regulation was further demonstrated by cell-based experiments involving *IRF1* overexpression in lung epithelial cells (***Fig. 3D***) and *IRF1* knockout in a respiratory epithelial cell line (***Fig. 3E***). Moreover, ChIP-seq showed a sharp peak in the upstream region of *HLA-F*, indicating direct binding and transcriptional regulation of *IRF1* on *HLA-F* (***Fig. 3F***).

Furthermore, mediation analysis revealed that *HLA-F* mediated the protective effects of *IRF1* on acute physiology severity (*β*_indirect_ = −4.50, 95% CI: −7.31 to −1.69, 98.9% mediated, *P =* 0.002), ARDS risk (*β*_indirect_ = −0.42, 95% CI: −0.69 to −0.15, 77.1% mediated, *P =* 0.002), and both 28-day (*β*_indirect_ = −0.53, 95% CI: −0.99 to −0.08, 73.8% mediated, *P =* 0.022) and 60-day survival (*β*_indirect_ = −0.47, 95% CI: −0.88 to −0.07, 77.8% mediated, *P =* 0.022; ***Fig. 3G*** and ***3H***), which implies a potential regulatory pathway.

### Early activation of *IRF1* benefits clinical outcomes, especially for severe patients

**Figure 4:**
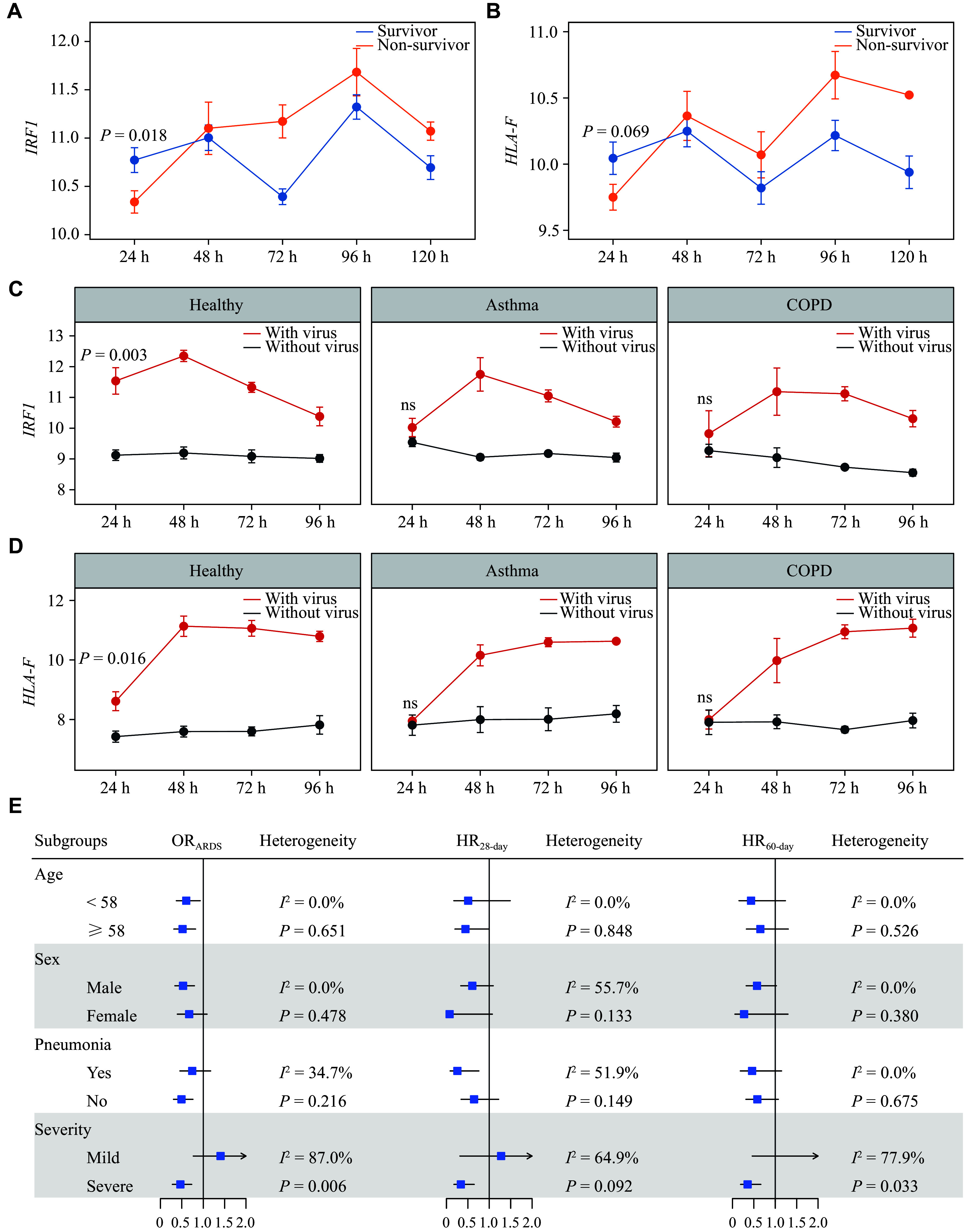
Time-series RNA expression and subgroup analyses. A and B: Time-series RNA expression for *IRF1* and *HLA-F* among survivor and non-survivor sepsis patients. C and D: Time-series RNA expression profiles of virus-infected airway epithelial cells derived from healthy, asthma, and COPD participants. E: Heterogeneity across participant subgroups (defined by age, sex, pneumonia, and disease severity) was evaluated through stratified analyses. Abbreviations: ARDS, acute respiratory distress syndrome; COPD, chronic obstructive pulmonary disease; HLA-F, human leukocyte antigen F; HR, hazard ratio; IRF1, interferon regulatory factor 1; OR, odds ratio.

Immunosuppression is a common disorder in sepsis patients, leading to poor clinical outcomes. Time-series RNA expression data showed that, among critically ill sepsis patients, *IRF1* and *HLA-F* were activated earlier in survivors, whereas early immunity was suppressed in non-survivors (***Fig. 4A*** and ***4B***). Further *in vitro* experiments in airway epithelial cell infection models from healthy, asthma, and COPD revealed that *IRF1* and *HLA-F* were rapidly activated within 24 h after viral infection in cells from healthy participants. Compared with the cells from healthy participants, those from asthma and COPD patients exhibited immunosuppression characterized by delayed and reduced immune responses (***Fig. 4C*** and ***4D***). These results indicate that early activation of the *IRF1-HLA* pathway in critically ill sepsis patients may play a prophylactic role in reducing ARDS susceptibility and mortality; however, whether preventive intervention would benefit all sepsis patients remains unclear. Subgroup analyses revealed heterogeneous effects among patients with different APACHE scores, suggesting that early prophylactic activation of *IRF1* may preferentially benefit patients with severe sepsis but not those with mild sepsis (***Fig. 4E***).

## Discussion

To our knowledge, this work may be the first to comprehensively evaluate the causal associations between IFN-related genes and sepsis-associated ARDS in large-scale populations. We developed a rigorous three-step, multi-cohort study design that allowed us to demonstrate that *IRF1* robustly contributes to both ARDS risk and survival. Mediation analyses and biological experiments further revealed that *IRF1* might play a crucial role in the immune response and affect ARDS development by targeting the MHC.

Substantial heterogeneity in prognosis has been observed among ARDS patients with similar clinical characteristics, which hampers effective treatment and underscores the importance of understanding the molecular mechanisms^[2,36]^. Previous studies have linked several molecular biomarkers to ARDS, but have failed to establish causality, thereby limiting their clinical applications. The current study addressed these limitations using TWAS, which employs instrumental variable techniques to infer causal relationships between gene expression and ARDS and identify relevant biomarkers for future clinical trials.

Mechanistically, *IRF1* can rapidly and dynamically respond to infections and suppress viral replication^[12]^. The basal expression of *IRF1* contributes to resistance against multiple viral infections and chronic inflammation^[37]^. Immunosuppression is a common disorder in sepsis patients, resulting in failure to eradicate primary infections and an increased susceptibility to lethal secondary infections. Notably, *IRF1* has been shown to stimulate and activate immune cells and restore antigen presentation capacity^[38]^. A cohort study has suggested that *IRF1* is associated with the risk of asthma^[39]^. Furthermore, emerging evidence indicates the importance of the timing of IFN signaling activation during host infection: early activation is associated with rapid improvement in immune responses, while delayed activation may lead to inflammation and pneumonia. This is consistent with our findings, in which higher *IRF1* expression at ICU admission is associated with reduced ARDS risk and improved survival among sepsis patients. Our time-series transcriptional data also demonstrated suppressed and delayed immune responses in respiratory cells from asthma and COPD patients, as well as in sepsis patients with poor outcomes. Nevertheless, excessive immune responses may lead to prolonged immunosuppression and an increased risk of opportunistic infection^[40]^. A delayed and prolonged immune response was observed in non-survivors with sepsis.

In the pilot experimental phase, we investigated the potential roles of *IRF1*-MHC signaling using the Rhinovirus 1A-cultured airway epithelial cells, asthma models, and COPD models, considering the potential shared pathophysiological mechanisms between these experimental models and sepsis-associated ARDS^[41]^. Specifically, the activation of IFN and MHC pathways is conserved across diverse respiratory pathologies, including sepsis-induced lung injury^[41–42]^. Furthermore, patients with asthma or COPD exhibit heightened susceptibility to ARDS during acute exacerbations or infections, making these models biologically relevant for exploring shared molecular drivers. Nevertheless, further direct experimental evidence from ARDS models is warranted to validate the "*IRF1*→*HLA-F*→ARDS" axis. Our findings suggest that early prophylactic interventions to activate *IRF1* might be promising for reducing the risk of ARDS development and mortality, particularly in severely ill sepsis patients.

In addition to regulating IFN signaling, *IRF1* can directly activate immune responses and engage in antiviral responses^[15]^. Our findings indicate that *IRF1* is widely and strongly coexpressed with MHC genes and influences ARDS risk and survival by regulating these genes, which are involved in antigen presentation and the activation of both innate and adaptive immunity^[43]^. We found that *HLA-F* was associated with acute physiological severity, ARDS risk and survival, and it significantly mediated the protective effect of *IRF1*. *HLA-F* is a non-classical molecule of MHC class Ⅰ, containing a distinct peptide-binding groove that can present diverse peptides^[44]^. *HLA-F* regulates immunity by interacting with natural killer cell receptors, engaging in antiviral responses, and reducing the risk and severity of respiratory failure^[45]^. Moreover, *HLA-F* is a high-affinity ligand of killer cell immunoglobulin-like receptors that are expressed in NK cells, CD4^+^ T cells, CD8^+^ T cells, and gamma-delta T cells^[45]^. These cells play crucial roles in the pathogenesis and prognosis of ARDS^[46–47]^.

As a master regulator of IFN signaling and immune homeostasis, *IRF1* presents a promising yet dual-edged target for therapeutic intervention of sepsis-associated ARDS. On the one hand, *IRF1* can enhance immune responses, inhibit viral replication, and reduce bacterial infections by regulating certain anti-inflammatory factors^[48]^. On the other hand, during the acute phase of ARDS, *IRF1* can activate the expression of proinflammatory cytokines, such as TNF-α and IL-6, thereby aggravating lung inflammation. *IRF1* functions within a complex regulatory network, exhibiting context-dependent roles as both a pro-survival mediator in early infection and a pro-apoptotic agent in chronic inflammation. This necessitates careful optimization of therapeutic dose and timing^[49]^. Future studies should explore optimal treatment windows, combinatorial strategies, and conditional activation approaches to maximize therapeutic efficacy.

The current study presents several strengths. First, we systematically evaluated the associations between IFN-related genes and both ARDS risk and survival. We identified a causal pathway, *IRF1*→*HLA-F*→ARDS, which may provide novel drug targets for immune prevention and therapy of sepsis-associated ARDS. Second, we integrated large-scale genotype and gene expression data from three independent cohorts, enhancing the robustness of our analysis. Third, we employed TWAS leveraging instrumental variable techniques to infer the causal effects of the genes, strengthening causal influence beyond simple associations. The causal link between *IRF1* and ARDS highlights promising avenues for future clinical applications. Finally, our rigorous three-step design, which included independent validation and biological experiment, further enhanced the reliability and robustness of our findings.

We acknowledge several limitations of the current study. First, our study population consisted primarily of individuals of European ancestry, mainly because TWAS models for other ethnicities remain limited, which restricts the generalizability of our results to other ethnic populations. Second, the TWAS framework relies on GReX prediction, which requires robust eQTL data to infer causal relationships. Forty-one of the 64 genes were excluded because of insufficient eQTL evidence (*e.g.*, lack of tissue-specific models or significant *cis*-eQTLs). While this filtering improves result reliability, it may also lead to missed signals. Third, although the current study detected *IRF1* as a causal biomarker for prophylactic intervention, clinical trials are necessary to confirm the clinical utility of these findings.

In summary, the current study identified *IRF1* as a promising biomarker for sepsis-associated ARDS risk and survival, with these effects largely mediated by the downstream *HLA-F* gene. Our findings suggest that early prophylactic intervention to activate *IRF1* in sepsis patients may reduce the risk of ARDS development and mortality, especially among those with severe illness.

## Additional information

The online version contains supplementary material available at http://www.jbr-pub.org.cn/article/doi/10.7555/JBR.39.20250066.
