# Supplementary material for: Integrative omics and multi-cohort identify IRF1 and biological targets related to sepsis-associated acute respiratory distress syndrome
Source: J Biomed Res. 2025 May 27;40(1):11–22. doi: 10.7555/JBR.39.20250066 (PMC12794184; doi:10.7555/JBR.39.20250066)
Supplement: Supplementary file 1 — The online version contains supplementary material available at http://www.jbr-pub.org.cn/article/doi/10.7555/JBR.39.20250066. [file jbr-40-1-11-S1.pdf]

Available online at www.jbr-pub.org.cn

Open Access at PubMed Central

Journal of Biomedical Research, 2026 40(1): 1–11

Supplementary Data

JBR

Integrative omics and multi-cohort identify *IRF1* and biological targets related to sepsis-associated acute respiratory distress syndrome

Jiajin Chen[1,2,3,△], Ruili Hou[2,3,△], Xiaowen Xu[2,3,△], Ning Xie[2,3,△], Jiaqi Tang[2,3], Yi Li[4], Xiaoqing Nie[1], Nuala J. Meyer[5], Li Su[6], David C. Christiani[6,7,#], Feng Chen[2,3,✉], Ruyang Zhang[2,3,8,✉]

[1]*Institute of Cardiovascular Diseases, Xiamen Cardiovascular Hospital of Xiamen University, Xiamen University School of Medicine, Xiamen, Fujian 361006, China;*
[2]*Department of Biostatistics, Center for Global Health, School of Public Health, Nanjing Medical University, Nanjing, Jiangsu 211166, China;*
[3]*China International Cooperation Center for Environment and Human Health, Nanjing Medical University, Nanjing, Jiangsu 211166, China;*
[4]*Department of Biostatistics, University of Michigan, Ann Arbor, MI 48109, USA;*
[5]*Pulmonary, Allergy, and Critical Care Medicine Division, University of Pennsylvania Perelman School of Medicine, Philadelphia, PA 19104, USA;*
[6]*Department of Environmental Health, Harvard T.H. Chan School of Public Health, Boston, MA 02115, USA;*
[7]*Pulmonary and Critical Care Division, Department of Medicine, Massachusetts General Hospital and Harvard Medical School, Boston, MA 02114, USA;*
[8]*Changzhou Medical Center, Nanjing Medical University, Changzhou, Jiangsu 213164, China.*

Supplementary methods

GWAS data and quality control, and imputation

MEARDS

The MEARDS GWAS is part of the Identification of SNPs Predisposing to Altered Acute Lung Injury Risk (iSPAAR) consortium, which includes 40% acute respiratory distress syndrome (ARDS) cases and all at-risk controls. Participants were recruited at the intensive care units (ICUs) of MGH and BIDMC, Boston, Massachusetts between 1998 and 2014[1–2]. Briefly, eligible participants were critically ill patients with at least one predisposing condition for ARDS, including bacteremia, sepsis, septic shock, pneumonia, multiple fractures, pulmonary contusion, aspiration, or massive blood transfusion, and without any of the following exclusion criteria: age < 18 years, HIV infection, diffuse alveolar hemorrhage, chronic lung diseases other than chronic obstructive pulmonary disease or asthma, directive to withhold intubation, immunosuppression not secondary to corticosteroids, treatment with granulocyte colony-stimulating factor, cytotoxic therapy, or solid organ or bone marrow transplant. Data collected included demographics, medical history, vital signs, hematology, and chemistry. Frequent arterial blood gas analysis and chest radiography were performed within 24 h of ICU admission. All genotyped subjects were of European ancestry. DNA samples were genotyped using the Infinium HumanExome BeadChip (Illumina, Inc., San Diego, CA, USA), and standard quality control was performed for all iSPAAR GWAS samples. We excluded individuals with low call rates (< 95%), familial relationships, or extreme heterozygosity rates. We also excluded SNPs with minor allele frequencies

△These authors contributed equally to this work.
#Senior author who supervised this work.
✉Corresponding authors: Ruyang Zhang and Feng Chen, School of Public Health, Nanjing Medical University, 101 Longmian Avenue, Nanjing, Jiangsu 211166, China. E-mails: zhangruyang@njmu.edu.cn (Zhang) and fengchen@njmu.edu.cn (Chen).

Received: 22 February 2025; Revised: 20 May 2025; Accepted: 22 May 2025; Published online: 27 May 2025
CLC number: R563.8, Document code: A
The authors reported no conflict of interests.

This is an open access article under the Creative Commons Attribution (CC BY 4.0) license, which permits others to distribute, remix, adapt and build upon this work, for commercial use, provided the original work is properly cited.

© 2026 by Journal of Biomedical Research.

https://doi.org/10.7555/JBR.39.20250066

(MAF) < 1%, low call rates (< 95%), and $P < 1 \times 10^{-6}$ in Hardy-Weinberg equilibrium (HWE) tests. As a result, 27 418 SNPs were removed because of low MAF, four SNPs were removed because of low call rate, and 64 SNPs were removed because of deviation from HWE. Finally, a total of 374 sepsis-associated ARDS cases and 995 sepsis-only controls with 490 773 SNPs were included for analyses.

MESSI

The MESSI cohort was approved by the Institutional Review Board (IRB) of the University of Pennsylvania (IRB #808542). Participants in the MESSI GWAS cohort were recruited at the medical ICU of the Hospital of the University of Pennsylvania, an urban tertiary referral center, between 2008 and 2015[3–4]. Whole blood was collected for DNA, and plasma was collected within 24 h of ICU admission, as close to the time of ICU arrival as possible. Clinical data were abstracted from the electronic medical record. SNPs were genotyped using the Axiom TxArray v1 (Affymetrix, Santa Clara, CA, USA) and followed by standard quality control. We excluded individuals with low call rates (< 95%), familial relationships, or extreme heterozygosity rates. We also excluded SNPs with MAF < 1%, low call rates (< 95%), and $P < 1\times10^{-6}$ in HWE tests (164 403 SNPs were removed because of low MAF; 23 598 SNPs removed due to low call rate; 46 160 SNPs were removed because of deviation from HWE). Finally, a total of 603 participants of European ancestry with 531 589 SNPs were included for analyses.

GWAS imputation procedures

Whole genome imputation for chromosomes 1–22 was performed with Minimac3 using the 1000 Genome Project Phase 3, version 5 (n = 2 504) as the reference panel. The initial imputed dosages were filtered and converted to best-guess genotypes *via* qctool v2 and PLINK 1.9. Imputed SNPs with $P < 10^{-6}$ in the HWE test, imputation quality $r^2 < 0.8$, or MAF < 0.01 were excluded before association analysis.

RNA-array data from the MARS cohort

MARS is a prospective observational study in the mixed ICUs of two tertiary teaching hospitals (Academic Medical Center in Amsterdam and University Medical Center in Utrecht), between January 2011 and July 2013 (ClinicalTrials.gov identifier: NCT01905033)[5]. The MARS study was approved by the local ethics committee (IRB No. 10-056C). All consecutive patients above 18 years of age admitted between January 2011 and July 2013 with an expected length of stay longer than 24 h were included. MARS RNA-array is available in GEO under accession code GSE65682.

RNA-sequencing data of MEARDS

A total of 108 sepsis-associated ARDS cases and 105 sepsis-only controls were recruited for RNA-sequencing analysis from the MEARDS cohort[1]. These samples did not overlap with the samples in the MEARDS GWAS. Total RNA was extracted from the blood using the PAXgene Blood RNA Kit (Qiagen) and subjected to poly(A) selection by oligo(dT) beads. Subsequently, RNA libraries were prepared using the MGIEasy RNA Library Prep Kit according to the manufacturer's protocols. The libraries were then processed with high-throughput sequencing for pair-ends of 100 bp using the MGISEQ-2000 platform. Sequencing reads containing low-quality, adaptor-polluted, and high content of unknown base (N) reads were removed, and high-quality pair-end reads were retained as clean data. We collaborated with Harvard Chan Bioinformatics Core (HBC: https://bioinformatics.sph.harvard.edu/) for further downstream analyses. All samples were processed using the RNA-sequencing pipeline implemented in the bcbio-nextgen project (https://bcbio-nextgen.readthedocs.org/en/latest/). Briefly, clean reads were aligned and mapped to the reference genome GRCh38 using STAR[6], and counts of reads aligning to known genes were generated by featureCounts[7]. Alignments were checked for evenness of coverage, rRNA content, genomic context of alignments (*e.g.*, alignments in known transcripts and introns), complexity, and other quality checks using a combination of FastQC, Qualimap[8], MultiQC (https://github.com/ewels/MultiQC), and bcbioRNAseq (version 0.2.9: https://github.com/bcbio/bcbio-nextgen)[9] In parallel, Transcripts Per Million (TPM) measurements per isoform were generated by quasi-alignment using Salmon[10] and summarized at the gene level for differential expression analysis because quantitation at the isoform level produces more accurate results at the gene level[11]. Only participants with completed clinical data were included for analyses.

TWAS analysis and MASHR models

TWAS leverages instrumental variable techniques to infer causality between gene expressions and traits under the framework of Mendelian randomization. The traditional TWAS consists of three steps. First, prediction models of gene expressions and genetic

variants were constructed, based on the reference panel. Second, these models were used to predict gene expression in an individual GWAS cohort. Finally, the causal effects between the predicted gene expression and outcome were estimated (***Supplementary Fig. 8***, available online).

Numeric statistical methods were developed for training TWAS models. In the current study, we applied the MASHR-based models as recommended by the PrediXcan team (https://github.com/hakyimlab/MetaXcan/wiki)[12]. The MASHR method applies an empirical Bayesian method to smooth the *cis*-eQTL effect size estimates by leveraging the correlation between tissues. MASHR also provides a measure of significance for each effect in each tissue. Specifically, MASHR outputs the local false sign rate (LFSR)[13], which is the probability that the effect is estimated with the incorrect sign. The LFSR is analogous to the local false discovery rate[14], but is more stringent in that it insists that effects be correctly signed to be considered true discoveries. Eligible models were defined with a local false sign rate ≤ 0.05[12]. MASHR estimated the LFSR for each effect separately in each tissue.

The PrediXcan team trained the MASHR-based TWAS models based on GTEx v8 samples of European ancestry, which are publicly accessible from PredictDB (https://predictdb.org/). We downloaded the tissue-specific MASHR models of whole blood tissue and further predicted the genetically determined components of gene expression for interferon (IFN)-related genes under the framework of individual-level PrediXcan. A step-by-step official tutorial was also provided by the PrediXcan team (https://github.com/hakyimlab/MetaXcan/wiki/Individual-level-PrediXcan:-introduction,-tutorials-and-manual).

In silico analysis

PhenomeXcan (http://apps.hakyimlab.org/phenomexcan/) is a gene-based resource documenting the results of Phenome-wide association studies (PheWASs) for 22 255 genes across 4 091 traits (4 049 documented in the UK Biobank)[15].

Single-sample gene set enrichment analysis (ssGSEA)[16], an extension of Gene Set Enrichment

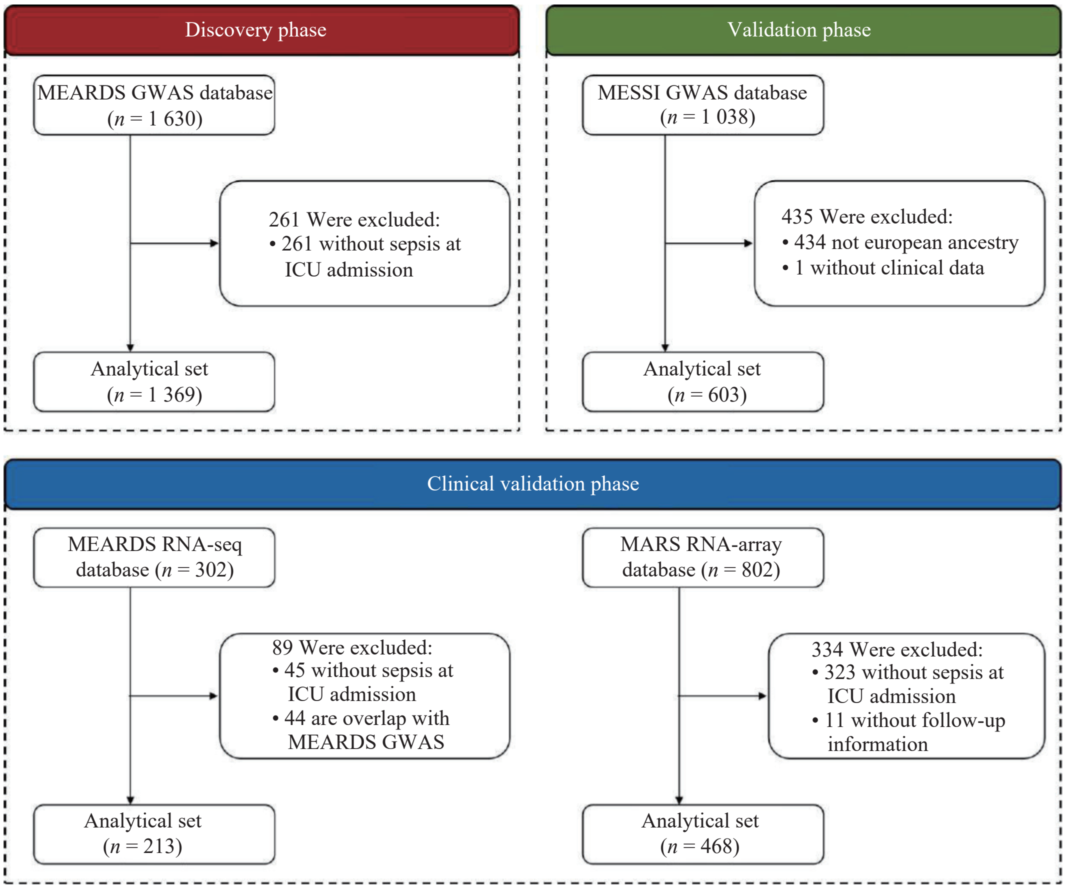

Supplementary Fig. 1 **The flow diagram of study samples.** Abbreviations: ARDS, acute respiratory distress syndrome; ICU, intensive care unit; GWAS, genome-wide association study; MARS, Molecular Diagnosis and Risk Stratification of Sepsis; MEARDS, Molecular Epidemiology of ARDS; MESSI, Molecular Epidemiology of Sepsis in the ICU.

Analysis (GSEA), calculates the separate scores for each sample for the gene sets. The ssGSEA enrichment score represents the degree to which the genes in a particular gene set are coordinately up- or down-regulated within a sample. The gene sets of pathways were obtained from the MSigDB (https://www.gsea-msigdb.org/gsea/msigdb/index.jsp), and the gene sets for 28 immune cells from 37 studies

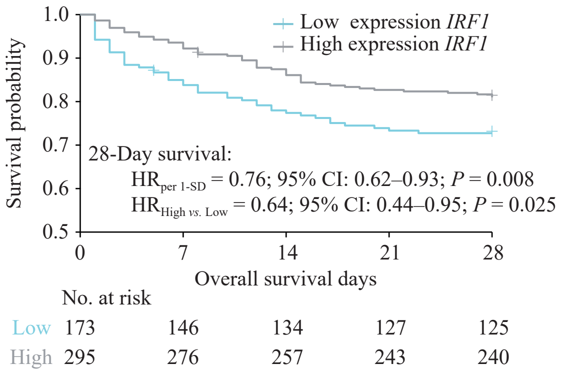

Supplementary Fig. 2 **Kaplan–Meier plot for survival difference of *IRF1* in MARS cohort.** Abbreviations: CI, confidence interval; HR, hazard ratio; IRF1, interferon regulatory factor 1; MARS, Molecular Diagnosis and Risk Stratification of Sepsis.

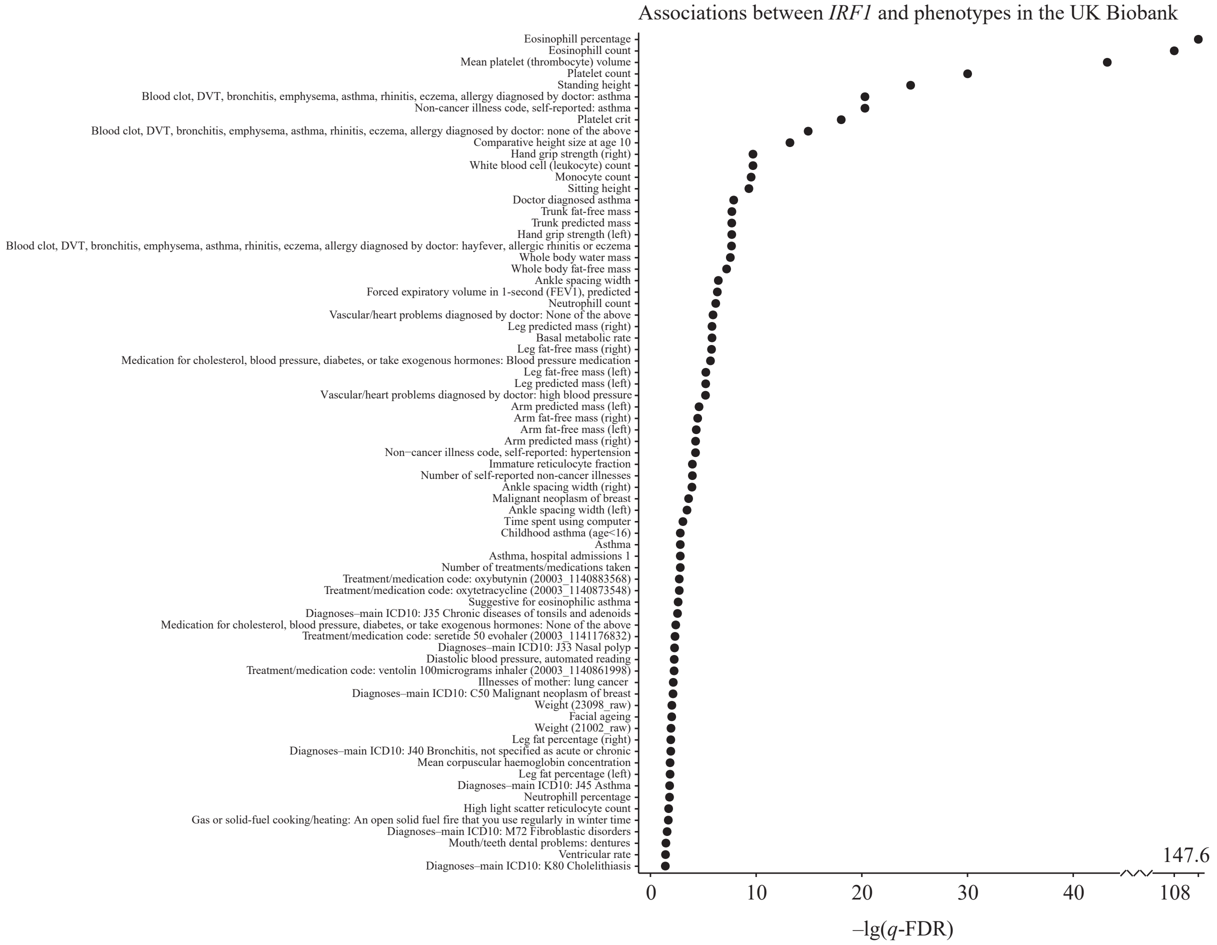

Supplementary Fig. 3 **Correlations between *IRF1* and multiple traits.** Analysis was conducted based on PhenomeXcan, which supported associations between gene expressions and 4 049 traits in the UK Biobank. Only traits with q-FDR ≤ 0.05 were displayed. Abbreviations: IRF1, interferon regulatory factor 1.

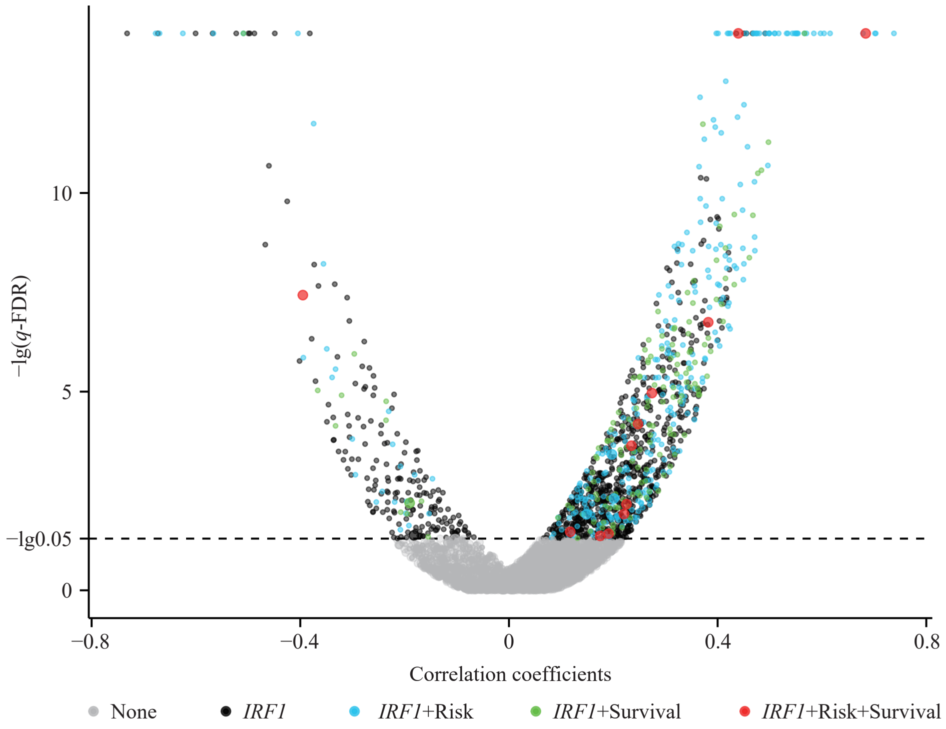

Supplementary Fig. 4 **Volcano plot for correlations between *IRF1* and enriched pathway.** The pathway enrichment levels for each sample were analyzed using the ssGSEA algorithm. The annotated gene sets were obtained from the MsigDB database. Abbreviation: IRF1, interferon regulatory factor 1.

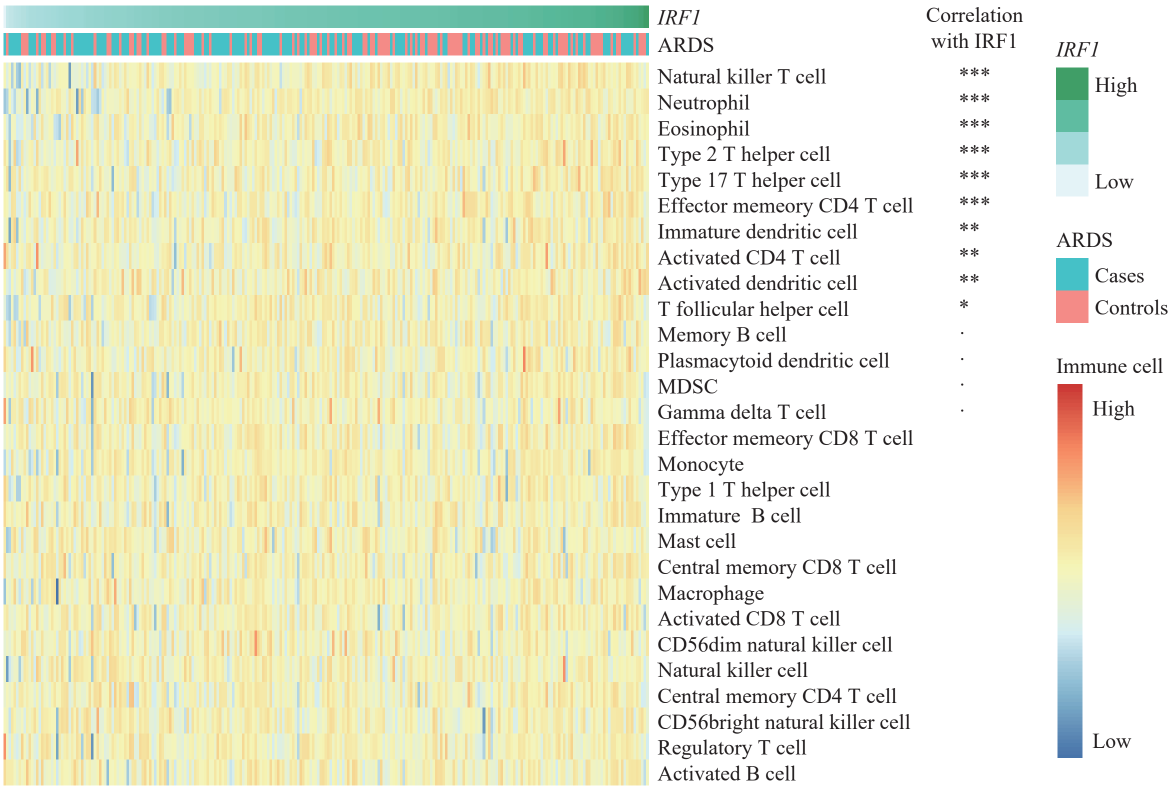

Supplementary Fig. 5 **Correlations between *IRF1* and immune cells.** The abundances of 28 immune cells were inferred by using ssGSEA. Correlations were evaluated by the Spearman method. $^{***}P \leq 0.001$, $^{**}P \leq 0.01$, $^{*}P \leq 0.05$, and $^{\cdot}P \leq 0.1$. Abbreviations: ARDS, acute respiratory distress syndrome; IRF1, interferon regulatory factor 1.

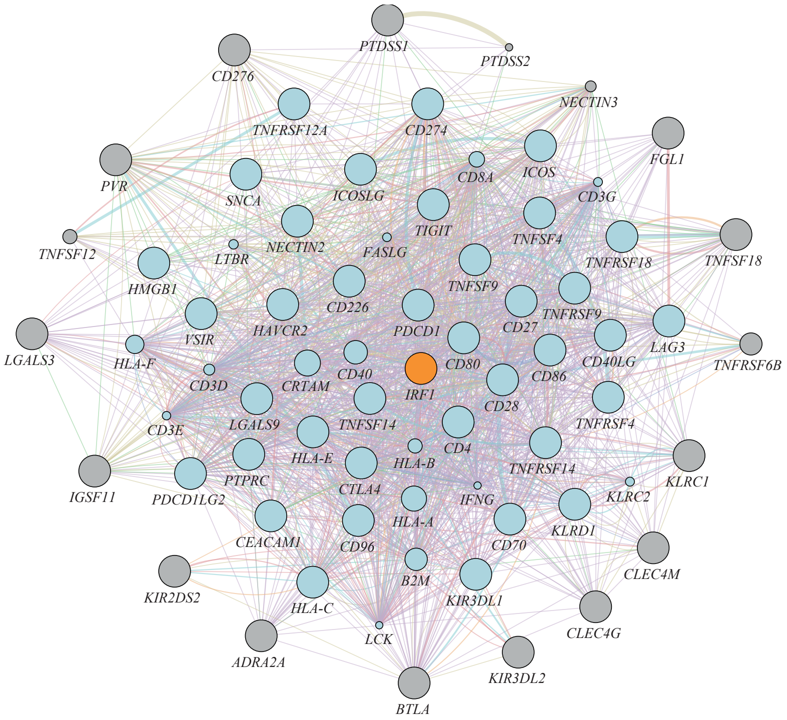

Supplementary Fig. 6 **Gene network for *IRF1* and immune checkpoint genes.** The network was conducted by GeneMINIA, a plugin of the Cytoscape application. The blue circles represent the first neighbors of *IRF1*, which have a direct functional relationship with *IRF1*. Abbreviation: IRF1, interferon regulatory factor 1.

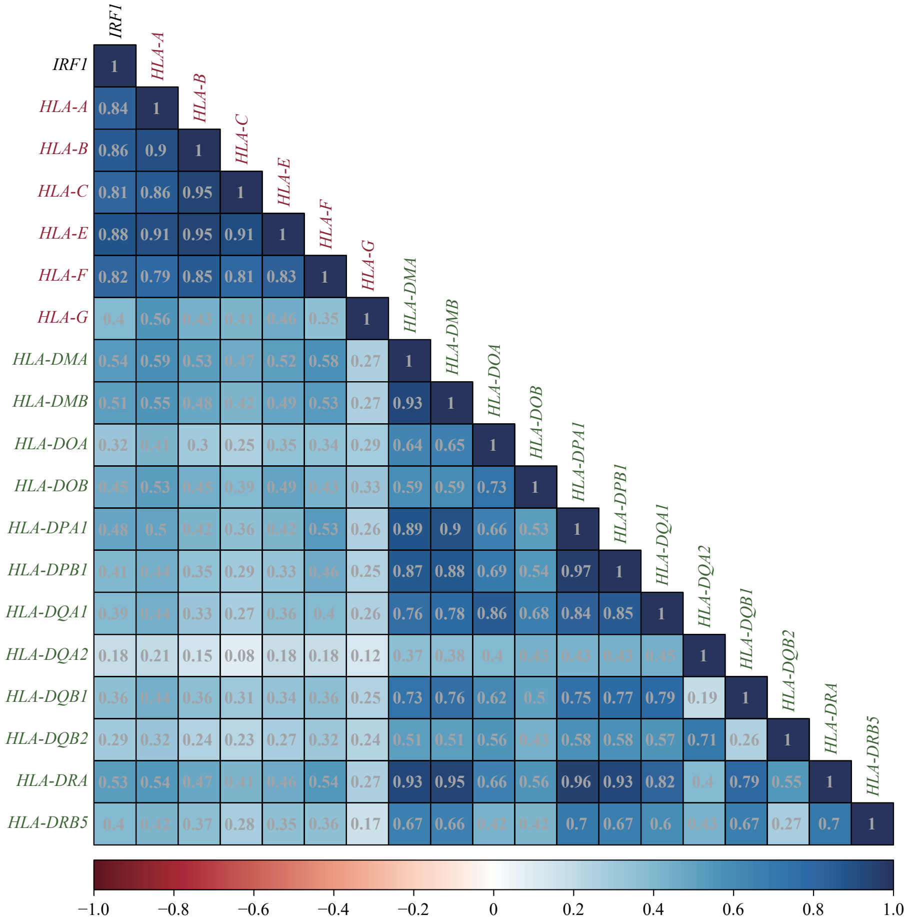

Supplementary Fig. 7 **Correlations between *IRF1* and major histocompatibility complex (MHC) genes.** The genes belonging to MHC class Ⅰ were highlighted in red fonts, while MHC class Ⅱ genes were highlighted in green.

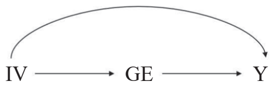

Supplementary Fig. 8 **The diagram of TWAS.** IV: instrumental variable; GE: gene expression; Y: outcome.

Supplementary Table 1 **Demographic and clinical characteristics in three study populations**

Characteristic	MEARDS GWAS			MESSI GWAS			MEARDS RNA-seq			MARS RNA-seq		
	ARDS (*n* = 374)	Non-ARDS (*n* = 995)	*P*	ARDS (*n* = 267)	Non-ARDS (*n* = 336)	*P*	ARDS (*n* = 108)	Non-ARDS (*n* = 105)	*P*	ARDS (*n* = 51)	Non-ARDS (*n* = 42)	*P*
Age	60.4 ± 16.9	63.4 ± 16.1	0.003	58.9 ± 14.1	61.5 ± 13.8	0.023	53.0 ± 18.4	60.6 ± 19.2	0.003	61.9 ± 12.0	62.2 ± 13.5	0.910
Sex			0.863			0.711			0.554			0.270
Male	227 (60.7)	597 (60.0)		163 (61.0)	199 (59.2)		65 (60.2)	58 (55.2)		27 (53.0)	27 (64.3)	
Female	147 (39.3)	398 (40.0)		104 (39.0)	137 (40.8)		43 (39.8)	47 (44.8)		24 (47.0)	15 (35.7)	
Pneumonia	282 (75.4)	471 (47.3)	2.47×10^{-20}				60 (55.6)	41 (39.0)	0.023	1 (2.0)	0	
Lung infection				166 (62.2)	134 (39.9)	8.51×10^{-8}						
Aspiration	30 (8.0)	60 (6.0)	0.229				14 (13.0)	3 (2.9)	0.014			
Transfer	16 (4.3)	22 (2.2)	0.059				11 (10.2)	3 (2.9)	0.060			
Trauma	7 (1.9)	5 (0.5)	0.036				2 (1.9)	3 (2.9)	0.975			
Transfusion	16 (4.3)	22 (2.2)	0.059				3 (1.9)	7 (6.7)	0.160			
APACHE Ⅲ	79.2 ± 22.8	66.5 ± 22.5	2.43×10^{-19}	86.8 ± 31.4	74.1 ± 28.1	2.09×10^{-6}	74.4 ± 26.1	64.0 ± 22.7	0.004			

Data are presented as mean ± standard deviation or *n* (%). All participants are critically ill patients with sepsis, and no overlap between MEARDS GWAS data and MEARDS RNA-seq data. APACHE indicates the Acute Physiology and Chronic Health Evaluation score.

Supplementary Table 2 **List of interferon (IFN)-related gene families and members searched in the HGNC database**

Family ID	Gene family	Ensembl ID	Symbol	Name	Type
1600	IFN-induced transmembrane protein domain containing	ENSG00000204314	*PRRT1*	Proline-rich transmembrane protein 1	Protein-coding gene
1600	IFN-induced transmembrane protein domain containing	ENSG00000167371	*PRRT2*	Proline-rich transmembrane protein 2	Protein-coding gene
1600	IFN-induced transmembrane protein domain containing	ENSG00000183379	*SYNDIG1L*	Synapse differentiation-inducing 1 like	Protein-coding gene
1600	IFN-induced transmembrane protein domain containing	ENSG00000142046	*TMEM91*	Transmembrane protein 91	Protein-coding gene
1598	IFN-induced transmembrane proteins	ENSG00000185885	*IFITM1*	IFN-induced transmembrane protein 1	Protein-coding gene
1598	IFN-induced transmembrane proteins	ENSG00000244242	*IFITM10*	IFN-induced transmembrane protein 10	Protein-coding gene
1598	IFN-induced transmembrane proteins	ENSG00000185201	*IFITM2*	IFN-induced transmembrane protein 2	Protein-coding gene
1598	IFN-induced transmembrane proteins	ENSG00000142089	*IFITM3*	IFN-induced transmembrane protein 3	Protein-coding gene
1598	IFN-induced transmembrane proteins	ENSG00000206013	*IFITM5*	IFN-induced transmembrane protein 5	Protein-coding gene
599	IFN receptors	ENSG00000142166	*IFNAR1*	IFN alpha and beta receptor subunit 1	Protein-coding gene
599	IFN receptors	ENSG00000159110	*IFNAR2*	IFN alpha and beta receptor subunit 2	Protein-coding gene
599	IFN receptors	ENSG00000027697	*IFNGR1*	IFN gamma receptor 1	Protein-coding gene
599	IFN receptors	ENSG00000159128	*IFNGR2*	IFN gamma receptor 2	Protein-coding gene
599	IFN receptors	ENSG00000185436	*IFNLR1*	IFN lambda receptor 1	Protein-coding gene
1743	IFN regulatory factors	ENSG00000125347	*IRF1*	IFN regulatory factor 1	Protein-coding gene
1743	IFN regulatory factors	ENSG00000137265	*IRF4*	IFN regulatory factor 4	Protein-coding gene
1743	IFN regulatory factors	ENSG00000128604	*IRF5*	IFN regulatory factor 5	Protein-coding gene
1743	IFN regulatory factors	ENSG00000117595	*IRF6*	IFN regulatory factor 6	Protein-coding gene
1743	IFN regulatory factors	ENSG00000185507	*IRF7*	IFN regulatory factor 7	Protein-coding gene
1743	IFN regulatory factors	ENSG00000140968	*IRF8*	IFN regulatory factor 8	Protein-coding gene
1743	IFN regulatory factors	ENSG00000213928	*IRF9*	IFN regulatory factor 9	Protein-coding gene

Supplementary Table 2 **List of interferon (IFN)-related gene families and members searched in HGNC database** (continued)

Family ID	Gene family	Ensembl ID	Symbol	Name	Type
598	IFNs	ENSG00000182393	*IFNL1*	IFN lambda 1	Protein-coding gene
598	IFNs	ENSG00000136244	*IL6*	Interleukin 6	Protein-coding gene
1600	IFN induced transmembrane protein domain containing	ENSG00000283758	*PMIS2*	PMIS2 transmembrane protein	Protein-coding gene
1600	IFN-induced transmembrane protein domain containing	ENSG00000283526	*PRRT1B*	Proline-rich transmembrane protein 1B	Protein-coding gene
1600	IFN-induced transmembrane protein domain containing	ENSG00000101463	*SYNDIG1*	Synapse differentiation-inducing 1	Protein-coding gene
1600	IFN-induced transmembrane protein domain containing	ENSG00000224982	*TMEM233*	Transmembrane protein 233	Protein-coding gene
1600	IFN-induced transmembrane protein domain containing	ENSG00000184811	*TRARG1*	Trafficking regulator of GLUT4 (SLC2A4) 1	Protein-coding gene
1598	IFN-induced transmembrane proteins	ENSG00000235821	*IFITM4P*	IFN-induced transmembrane protein 4 pseudogene	Pseudogene
1598	IFN-induced transmembrane proteins	ENSG00000215096	*IFITM8P*	IFN-induced transmembrane protein 8 pseudogene	Pseudogene
1598	IFN-induced transmembrane proteins	ENSG00000213275	*IFITM9P*	IFN-induced transmembrane protein 9 pseudogene	Pseudogene
1743	IFN regulatory factors	ENSG00000168310	*IRF2*	IFN regulatory factor 2	Protein-coding gene
1743	IFN regulatory factors	ENSG00000126456	*IRF3*	IFN regulatory factor 3	Protein-coding gene
598	IFNs	ENSG00000197919	*IFNA1*	IFN alpha 1	Protein-coding gene
598	IFNs	ENSG00000186803	*IFNA10*	IFN alpha 10	Protein-coding gene
598	IFNs	ENSG00000231195	*IFNA11P*	IFN alpha 11 pseudogene	Pseudogene
598	IFNs	ENSG00000235108	*IFNA12P*	IFN alpha 12 pseudogene	Pseudogene
598	IFNs	ENSG00000233816	*IFNA13*	IFN alpha 13	Protein-coding gene
598	IFNs	ENSG00000228083	*IFNA14*	IFN alpha 14	Protein-coding gene
598	IFNs	ENSG00000147885	*IFNA16*	IFN alpha 16	Protein-coding gene
598	IFNs	ENSG00000234829	*IFNA17*	IFN alpha 17	Protein-coding gene
598	IFNs	ENSG00000188379	*IFNA2*	IFN alpha 2	Protein-coding gene
598	IFNs	ENSG00000226393	*IFNA20P*	IFN alpha 20 pseudogene	Pseudogene
598	IFNs	ENSG00000137080	*IFNA21*	IFN alpha 21	Protein-coding gene
598	IFNs	ENSG00000224416	*IFNA22P*	IFN alpha 22 pseudogene	Pseudogene
598	IFNs	ENSG00000236637	*IFNA4*	IFN alpha 4	Protein-coding gene
598	IFNs	ENSG00000147873	*IFNA5*	IFN alpha 5	Protein-coding gene
598	IFNs	ENSG00000120235	*IFNA6*	IFN alpha 6	Protein-coding gene
598	IFNs	ENSG00000214042	*IFNA7*	IFN alpha 7	Protein-coding gene
598	IFNs	ENSG00000120242	*IFNA8*	IFN alpha 8	Protein-coding gene
598	IFNs	ENSG00000171855	*IFNB1*	IFN beta 1	Protein-coding gene
598	IFNs	ENSG00000111537	*IFNG*	IFN gamma	Protein-coding gene
598	IFNs	ENSG00000147896	*IFNK*	IFN kappa	Protein-coding gene
598	IFNs	ENSG00000183709	*IFNL2*	IFN lambda 2	Protein-coding gene
598	IFNs	ENSG00000197110	*IFNL3*	IFN lambda 3	Protein-coding gene
598	IFNs	ENSG00000230208	*IFNNP1*	IFN nu 1 pseudogene	Pseudogene
598	IFNs	ENSG00000177047	*IFNW1*	IFN omega 1	Protein-coding gene
598	IFNs	ENSG00000232281	*IFNWP15*	IFN omega 1 pseudogene 15	Pseudogene
598	IFNs	ENSG00000223684	*IFNWP18*	IFN omega 1 pseudogene 18	Pseudogene
598	IFNs	ENSG00000238271	*IFNWP19*	IFN omega 1 pseudogene 19	Pseudogene
598	IFNs	ENSG00000237691	*IFNWP2*	IFN omega 1 pseudogene 2	Pseudogene
598	IFNs	ENSG00000225027	*IFNWP4*	IFN omega 1 pseudogene 4	Pseudogene
598	IFNs	ENSG00000232138	*IFNWP5*	IFN omega 1 pseudogene 5	Pseudogene
598	IFNs	ENSG00000226597	*IFNWP9*	IFN omega 1 pseudogene 9	Pseudogene

We searched and identified all five gene families (containing 64 genes) directly related to IFN from the HGNC databases, including IFNs, IFN receptors, IFN regulatory factors, IFN-induced transmembrane protein domain containing, and IFN-induced transmembrane proteins. Then, all these genes were included in the further analysis.

Supplementary Table 3 **Associations between IFN-related genes and ARDS risk in the MEARDS cohort and the MESSI cohort**

Ensemble ID	Symbol	MEARDS				MESSI		
		OR	95% CI	*P*	*q*-FDR	OR	95% CI	*P*
ENSG00000185201	*IFITM2*	0.81	(0.72–0.92)	0.000 8	0.019 4	1.06	(0.89–1.25)	0.524 8
ENSG00000204314	*PRRT1*	0.83	(0.73–0.94)	0.003 3	0.038 1	0.99	(0.84–1.17)	0.935 0
ENSG00000125347	*IRF1*	0.84	(0.74–0.96)	0.007 8	0.041 4	0.83	(0.71–0.99)	0.034 0
ENSG00000185885	*IFITM1*	0.85	(0.75–0.96)	0.008 6	0.041 4	1.05	(0.89–1.24)	0.545 4
ENSG00000137265	*IRF4*	1.18	(1.04–1.33)	0.009 4	0.041 4	0.90	(0.76–1.07)	0.224 9
ENSG00000142089	*IFITM3*	1.18	(1.04–1.33)	0.010 8	0.041 4	0.91	(0.77–1.07)	0.262 6
ENSG00000159110	*IFNAR2*	0.90	(0.79–1.03)	0.121 3	0.398 7			
ENSG00000128604	*IRF5*	1.09	(0.96–1.23)	0.178 4	0.472 3			
ENSG00000206013	*IFITM5*	1.09	(0.96–1.24)	0.187 1	0.472 3			
ENSG00000185436	*IFNLR1*	0.92	(0.82–1.04)	0.205 3	0.472 3			
ENSG00000142046	*TMEM91*	1.08	(0.95–1.22)	0.263 9	0.551 8			
ENSG00000159128	*IFNGR2*	1.05	(0.92–1.20)	0.430 4	0.799 9			
ENSG00000136244	*IL6*	0.95	(0.84–1.08)	0.452 1	0.799 9			
ENSG00000027697	*IFNGR1*	1.04	(0.91–1.17)	0.581 6	0.921 2			
ENSG00000185507	*IRF7*	1.03	(0.91–1.17)	0.621 5	0.921 2			
ENSG00000213928	*IRF9*	1.03	(0.91–1.17)	0.640 8	0.921 2			
ENSG00000142166	*IFNAR1*	0.98	(0.87–1.11)	0.766 9	0.983 4			
ENSG00000140968	*IRF8*	0.99	(0.87–1.12)	0.887 4	0.983 4			
ENSG00000117595	*IRF6*	0.99	(0.88–1.12)	0.900 5	0.983 4			
ENSG00000167371	*PRRT2*	1.00	(0.89–1.14)	0.951 9	0.983 4			
ENSG00000183379	*SYNDIG1L*	1.00	(0.88–1.14)	0.963 4	0.983 4			
ENSG00000244242	*IFITM10*	1.00	(0.88–1.13)	0.968 2	0.983 4			
ENSG00000182393	*IFNL1*	1.00	(0.88–1.13)	0.983 4	0.983 4			

Supplementary Table 4 **Associations between *IRF1*-correlated pathways and ARDS**

Pathway	Correlation with IRF1		Association with ARDS risk		Association with ARDS 28-day survival		Association with ARDS 60-day survival	
	r	*P*-value	*β*	*P*-value	*β*	*P*-value	*β*	*P*-value
Maturation of SARS-CoV-1 nucleoprotein	0.69	2.00E−16	−74.40	0.043 7	−82.50	0.015 7	−69.83	0.035 8
Type Ⅱ IFN signaling pathways	0.50	2.00E−16	−33.65	0.008 6	−29.18	0.033 6	−25.30	0.049 2
Ligand-independent caspase activation	0.45	2.00E−16	−109.12	0.004 4	−63.10	0.011 1	−47.30	0.052 6
DNA damage recognition in GG-NER	−0.54	2.00E−16	98.80	0.024 6	98.24	0.039 1	91.01	0.040 3
Regulation of PTEN localization	0.53	2.00E−16	−165.67	0.010 8	−134.60	0.023 9	−136.97	0.015 5
Heme synthesis	−0.62	2.00E−16	29.39	0.009 1	20.02	0.036 4	15.81	0.062 6
Odorant transduction	0.39	9.70E−11	−42.02	0.036 1	−52.00	0.045 9	−54.65	0.021 3
RHOT1 GTPase cycle	−0.37	1.91E−09	52.08	0.033 9	74.21	0.005 4	60.02	0.011 8
TRIF mediated programmed cell death	0.35	1.09E−08	−71.69	0.026 5	−43.54	0.016 8	−38.54	0.032 8
SARS-CoV infections	0.30	1.22E−06	−136.13	0.021 9	−163.44	0.008 5	−136.21	0.014 8
Cysteine and methionine metabolism	−0.27	1.08E−05	34.42	0.022 6	37.05	0.004 4	30.55	0.010 5
Olfactory signaling pathway	0.24	9.29E−05	−25.52	0.043 9	−44.37	0.003 0	−39.26	0.004 6
Hedgehog-GLI pathway	0.21	0.000 7	−60.52	0.013 9	−47.72	0.016 9	−41.74	0.020 5
RHO GTPases activate PAKs	0.19	0.002 1	−50.90	0.005 0	−37.86	0.041 5	−32.99	0.056 1
PKCγ Calcium-signaling pathway	0.19	0.002 4	−17.42	0.031 5	−20.02	0.008 4	−20.46	0.003 1
Potential therapeutics for SARS	0.15	0.018 7	−90.26	0.032 4	−113.41	0.007 4	−83.73	0.038 1

Supplementary Table 5 **Potential targeted drugs for *IRF1* derived from the DrugBank database**

Index	Drug name	Target gene	Drug group	Change
1	Alitretinoin	*IRF1*	Approved Investigational	Upregulated
2	Amiodarone	*IRF1*	Approved Investigational	Upregulated
3	Arsenic trioxide	*IRF1*	Approved Investigational	Both
4	Acetylsalicylic acid	*IRF1*	Approved Vet Approved	Upregulated
5	Calcitriol	*IRF1*	Approved Nutraceutical	Downregulated
6	Cyclosporine	*IRF1*	Approved Investigational Vet Approved	Upregulated
7	Dacarbazine	*IRF1*	Approved Investigational	Upregulated
8	Decitabine	*IRF1*	Approved Investigational	Upregulated
9	Dinitrochlorobenzene	*IRF1*	Investigational	Upregulated
10	Formaldehyde	*IRF1*	Approved Vet Approved	Upregulated
11	Fenretinide	*IRF1*	Investigational	Upregulated
12	Methotrexate	*IRF1*	Approved	Upregulated
13	Silicon dioxide	*IRF1*	Approved	Upregulated
14	Silver	*IRF1*	Approved Investigational	Downregulated
15	Tamibarotene	*IRF1*	Investigational	Upregulated
16	Titanium dioxide	*IRF1*	Approved	Upregulated
17	Tretinoin	*IRF1*	Approved Investigational Nutraceutical	Upregulated
18	Troglitazone	*IRF1*	Approved Investigational Withdrawn	Upregulated
19	Vincristine	*IRF1*	Approved Investigational	Upregulated

Supplementary Table 6 **Associations between MHC genes and *IRF1*, acute physiologic severity, ARDS risk and survival**

Gene	Acute physiologic severity				ARDS risk				28-day survival				60-day survival			
	β	95% CI	P	q-FDR	OR	95% CI	P	q-FDR	HR	95% CI	P	q-FDR	HR	95% CI	P	q-FDR
HLA-A	−1.64	(−4.95, 1.67)	0.333	0.375	0.70	(0.52, 0.95)	0.021	0.048	0.29	(0.15, 0.55)	1.76E−04	0.003	0.40	(0.23, 0.69)	0.001	0.017
HLA-B	−2.55	(−5.83, 0.73)	0.129	0.193	0.75	(0.55, 1.02)	0.065	0.099	0.39	(0.22, 0.67)	7.47E−04	0.005	0.51	(0.32, 0.81)	0.004	0.019
HLA-C	−1.69	(−4.94, 1.56)	0.308	0.370	0.80	(0.60, 1.08)	0.146	0.176	0.43	(0.25, 0.72)	0.002	0.007	0.51	(0.33, 0.82)	0.005	0.019
HLA-E	−2.77	(−6.05, 0.50)	0.099	0.162	0.74	(0.54, 1.00)	0.048	0.086	0.43	(0.25, 0.75)	0.003	0.011	0.49	(0.30, 0.81)	0.005	0.019
HLA-F	−5.23	(−8.42, −2.04)	0.002	0.005	0.58	(0.42, 0.80)	0.001	0.006	0.41	(0.24, 0.69)	8.23E−04	0.005	0.48	(0.30, 0.77)	0.002	0.018
HLA-G	1.85	(−1.48, 5.17)	0.278	0.357	0.92	(0.69, 1.22)	0.565	0.598	0.42	(0.21, 0.82)	0.011	0.034	0.70	(0.42, 1.19)	0.188	0.308
HLA-DMA	−4.67	(−8.05, −1.29)	0.007	0.017	0.65	(0.47, 0.89)	0.007	0.017	0.63	(0.32, 1.24)	0.182	0.267	0.71	(0.40, 1.25)	0.232	0.349
HLA-DMB	−6.37	(−9.76, −2.97)	3.09E−04	0.001	0.58	(0.42, 0.80)	0.001	0.006	0.57	(0.28, 1.15)	0.117	0.234	0.64	(0.35, 1.19)	0.162	0.308
HLA-DOA	−4.80	(−8.25, −1.35)	0.007	0.017	0.80	(0.59, 1.08)	0.141	0.176	0.93	(0.56, 1.53)	0.766	0.811	0.85	(0.54, 1.32)	0.467	0.600
HLA-DOB	−0.04	(−3.56, 3.47)	0.980	0.980	0.96	(0.71, 1.30)	0.794	0.794	0.65	(0.36, 1.15)	0.140	0.234	0.67	(0.40, 1.12)	0.126	0.284
HLA-DPA1	−7.31	(−10.71, −3.91)	3.87E−05	4.73E−04	0.58	(0.42, 0.81)	0.001	0.006	0.54	(0.29, 1.02)	0.057	0.128	0.58	(0.33, 1.02)	0.059	0.152
HLA-DPB1	−7.19	(−10.59, −3.78)	5.25E−05	4.73E−04	0.61	(0.44, 0.84)	0.002	0.008	0.62	(0.33, 1.17)	0.143	0.234	0.67	(0.38, 1.19)	0.172	0.308
HLA-DQA1	−4.53	(−7.95, −1.12)	0.010	0.020	0.75	(0.56, 1.02)	0.066	0.099	1.15	(0.65, 2.04)	0.639	0.767	1.08	(0.66, 1.79)	0.757	0.833
HLA-DQA2	−0.52	(−4.00, 2.97)	0.772	0.817	0.79	(0.58, 1.06)	0.117	0.162	0.74	(0.40, 1.37)	0.338	0.435	0.87	(0.52, 1.47)	0.600	0.720
HLA-DQB1	−5.33	(−8.74, −1.91)	0.003	0.008	0.70	(0.52, 0.96)	0.028	0.056	1.10	(0.63, 1.92)	0.727	0.811	1.05	(0.66, 1.69)	0.833	0.833
HLA-DQB2	−2.40	(−5.82, 1.01)	0.169	0.235	0.84	(0.62, 1.12)	0.230	0.259	0.44	(0.22, 0.88)	0.019	0.050	0.51	(0.28, 0.91)	0.024	0.072
HLA-DRA	−6.44	(−9.81, −3.06)	2.50E−04	0.001	0.59	(0.43, 0.82)	0.001	0.006	0.64	(0.33, 1.25)	0.193	0.267	0.71	(0.40, 1.27)	0.252	0.349
HLA-DRB5	−4.34	(−7.76, −0.92)	0.014	0.025	0.64	(0.47, 0.87)	0.004	0.013	0.93	(0.52, 1.67)	0.817	0.817	1.05	(0.65, 1.72)	0.833	0.833

Abbreviations: ARDS, acute respiratory distress syndrome; CI, confidence interval; FDR, false discovery rate; HR, hazard ratio; OR, odds ratio.

were obtained from The Cancer Imaging Archive (TCIA)[17].

GeneMINIA is a Cytoscape plugin that predicts and provides visualization of the potential functional connection between favorite genes and gene sets (https://apps.cytoscape.org/apps/genemania).

Statistical analysis

The nonlinear relationship was assessed using spline regression, implemented using the R package *rms*. Subgroup analyses were conducted on strata with a sub-sample size greater than 30, and the median value was used to classify the continuous variables.

References

[1] Zhang R, Wang Z, Tejera P, et al. Late-onset moderate to severe acute respiratory distress syndrome is associated with shorter survival and higher mortality: A two-stage association study[J]. *Intensive Care Med*, 2017, 43(3): 399–407.

[2] Wei Y, Tejera P, Wang Z, et al. A missense genetic variant in *LRRC16A/CARMIL1* improves acute respiratory distress syndrome survival by attenuating platelet count decline[J]. *Am J Respir Crit Care Med*, 2017, 195(10): 1353–1361.

[3] Reilly JP, Wang F, Jones TK, et al. Plasma angiopoietin-2 as a potential causal marker in sepsis-associated ARDS development: Evidence from Mendelian randomization and mediation analysis[J]. *Intensive Care Med*, 2018, 44(11): 1849–1858.

[4] Guillen-Guio B, Lorenzo-Salazar JM, Ma SF, et al. Sepsis-associated acute respiratory distress syndrome in individuals of European ancestry: A genome-wide association study[J]. *Lancet Respir Med*, 2020, 8(3): 258–266.

[5] Scicluna BP, van Vught LA, Zwinderman AH, et al. Classification of patients with sepsis according to blood genomic endotype: A prospective cohort study[J]. *Lancet Respir Med*, 2017, 5(10): 816–826.

[6] Dobin A, Davis CA, Schlesinger F, et al. STAR: Ultrafast universal RNA-seq aligner[J]. *Bioinformatics*, 2013, 29(1): 15–21.

[7] Liao Y, Smyth GK, Shi W. featureCounts: An efficient general purpose program for assigning sequence reads to genomic features[J]. *Bioinformatics*, 2014, 30(7): 923–930.

[8] García-Alcalde F, Okonechnikov K, Carbonell J, et al. Qualimap: Evaluating next-generation sequencing alignment data[J]. *Bioinformatics*, 2012, 28(20): 2678–2679.

[9] Steinbaugh MJ, Pantano L, Kirchner RD, et al. bcbioRNASeq: R package for bcbio RNA-seq analysis[J]. *F1000Research*, 2018, 6: 1976.

[10] Patro R, Duggal G, Love MI, et al. Salmon provides fast and bias-aware quantification of transcript expression[J]. *Nat Methods*, 2017, 14(4): 417–419.

[11] Soneson C, Love MI, Robinson MD. Differential analyses for RNA-seq: Transcript-level estimates improve gene-level inferences[J]. *F1000Res*, 2015, 4: 1521.

[12] Urbut SM, Wang G, Carbonetto P, et al. Flexible statistical methods for estimating and testing effects in genomic studies with multiple conditions[J]. *Nat Genet*, 2019, 51(1): 187–195.

[13] Stephens M. False discovery rates: A new deal[J]. *Biostatistics*, 2017, 18(2): 275–294.

[14] Chen MH, Ibrahim JG, Chi YY. A new class of mixture models for differential gene expression in DNA microarray data[J]. *J Stat Plan Inference*, 2008, 138(2): 387–404.

[15] Pividori M, Rajagopal PS, Barbeira A, et al. PhenomeXcan: mapping the genome to the phenome through the transcriptome[J]. *Sci Adv*, 2020, 6(37): eaba2083.

[16] Barbie DA, Tamayo P, Boehm JS, et al. Systematic RNA interference reveals that oncogenic *KRAS*-driven cancers require TBK1[J]. *Nature*, 2009, 462(7269): 108–112.

[17] Charoentong P, Finotello F, Angelova M, et al. Pan-cancer Immunogenomic analyses reveal genotype-immunophenotype relationships and predictors of response to checkpoint blockade[J]. *Cell Rep*, 2017, 18(1): 248–262.
